# The expression profile of *Aedes albopictus* miRNAs is altered by dengue virus serotype-2 infection

**DOI:** 10.1186/s13578-015-0009-y

**Published:** 2015-04-16

**Authors:** Yanxia Liu, Yanhe Zhou, Jinya Wu, Peiming Zheng, Yiji Li, Xiaoying Zheng, Santhosh Puthiyakunnon, Zhijian Tu, Xiao-Guang Chen

**Affiliations:** Key Laboratory of Prevention and Control for Emerging Infectious Diseases of Guangdong Higher Institutes, Department of Pathogen Biology, School of Public Health and Tropical Medicine, Southern Medical University, Guangzhou, Guangdong P.R. China; Department of Parasitology, School of Medicine, Sun Yat-Sen University, Guangzhou, Guangdong P.R. China; Department of Biochemistry, Virginia Tech, Blacksburg, Virginia USA

**Keywords:** miRNA, Dengue virus, *Aedes albopictus*, Host-pathogen interaction

## Abstract

**Background:**

*Aedes albopictus* is an important vector of Dengue virus (DENV) and it has quickly invaded the tropical and temperate environments worldwide. A few studies have shown that, microRNAs (miRNAs) regulate mosquito defense against pathogens. However, there is no systematic analysis of the impact of DENV infection on miRNA expression in *Ae. albopictus*. We conducted this study to investigate the miRNA expression of *Ae. albopictus* upon DENV-2 infection using Illumina RNA sequencing.

**Results:**

A total of 103 known and 5 novel candidate miRNAs were identified in DENV-2 infected and non-infected adult female *Ae. albopictus*. Comparative analysis indicated that 52 miRNAs were significantly down-regulated and 18 were up-regulated significantly after infection. Furthermore, RT-qPCR validated the expression patterns of eleven of these differentially expressed miRNAs. Targets prediction and functional analysis of these regulated miRNAs suggested that miR-34-5p and miR-87 might be involved in the anti-pathogen and immune responses.

**Conclusion:**

This is the first systematic study on the impact of DENV infection on miRNA expression in *Ae. albopictus*. Complex changes in miRNA expression suggest a potential role of miRNAs in antiviral responses by regulating immune-related genes. This investigation provides information concerning DENV-induced miRNAs and offers clues for identifying potential candidates for vector based antiviral strategies.

**Electronic supplementary material:**

The online version of this article (doi:10.1186/s13578-015-0009-y) contains supplementary material, which is available to authorized users.

## Background

Dengue fever is among the most important global arboviral diseases of humans. An estimated 50 million human cases of dengue virus (DENV) infections occur annually, and over 40% of the world's population is at risk of infection [1]. Currently, there is no effective chemotherapy, specific treatment or effective vaccine for DENV infection owing to safety concerns and potential antibody-dependent enhancement. Therefore, the only effective way to prevent or control dengue transmission is to control the vector mosquito. *Aedes albopictus* (*Ae. albopictus*) is an important vector of DENV and is an important invasive species that quickly and aggressively spreads worldwide in tropical and temperate environments [1-3].

MicroRNAs (miRNAs) have been shown to regulate apoptosis, viral infection and other critical biological events in animals and plants [4-7]. In mosquitoes, a large number of miRNAs have been reported to exhibit altered profiles during infection and regulate the host immune responses [8-12]. Knocking down the Dicer1 and Ago1 mRNAs in *Anopheles* leads to increased sensitivity to *Plasmodium* infection, suggesting that miRNAs might be involved in defense reactions [11]. Moreover, *Wolbachia* uses aae-miR-2940 to regulate a methyltransferase gene for blocking DENV replication [13,14]. In addition, aga-miR-2304, aga-miR-2390 and aae-miR-375 have been identified to be involved in the modulation of host immune response in mosquitoes, and aae-miR-375 enhances DENV-2 infection in an *Aedes aegypti* (*Ae. aegypti*) cell line [15,16]. These studies indicate that mosquito miRNAs can positively or negatively modulate the host response to pathogen infection.

However, although *Ae. albopictus* is an important vector of DENV, the relationship between *Ae. albopictus* miRNAs and DENV infection remains unknown. In this study, to gain a better understanding of DENV infection in *Ae. albopictus* at the miRNA level, we performed deep sequencing of small RNAs in *Ae. albopictus* upon DENV-2 infection. We also comparatively analyzed the differentially expressed miRNAs between DENV-infected and control mosquitoes. Furthermore, we predicted the targets of these regulated miRNAs to investigate the roles of these miRNAs during DENV infection in *Ae. albopictus*.

## Results

### High-throughput sequencing and annotation of small RNAs in *Ae. albopictus*

**Figure 1:**
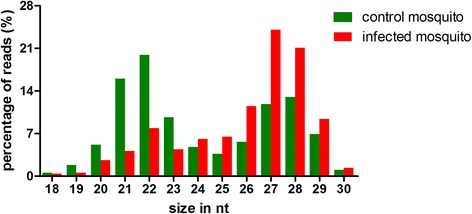
Length distribution of clean reads from deep sequencing in mosquitoes. The x-axis indicates sequence lengths from 18 nt to 30 nt. The y-axis indicates the percentage of reads for sequences of each length.

To evaluate the impact of DENV infection on miRNA expression in *Ae. albopictus*, we performed Illumina sequencing on small RNA libraries obtained from DENV-2 infected mosquitoes and compared it with uninfected mosquitoes. Approximately 10 and 20 million reads were obtained from control and infected mosquitoes, respectively. After removal of adaptors, contaminants and low quality reads, the length distribution of clean reads was summarized in Figure 1. We found that the peak in the control was at 21–22 nt with a percentage of 35.79; however, the peak of the infected sample was at 27–28 nt with a percentage of 44.82. As shown in Table 1, 4,805,249 clean reads from control and 3,309,844 clean reads from infected mosquitoes were mapped to *Ae. aegypti* genome using SOAP. Intron, exon, tRNA, rRNA, miRNA, snRNA and snoRNA reads were annotated, respectively.

**Table 1:** **Summary of sRNAs from control and DENV-infected** ***Aedes albopictus***

	**Control mosquitoes**		**Infected mosquitoes**	
**RNA class**	**Unique sRNAs**	**Total sRNAs**	**Unique sRNAs**	**Total sRNAs**
Raw reads		10931852		21966367
clean reads	1962591 (100%)	10102019 (100%)	5600491 (100%)	20781374 (100%)
Mapped to genomic	61337 (3.13%)	4805249 (47.57%)	123126 (2.20%)	3309844 (15.93%)
Exon antisense	20663 (1.06%)	71989 (0.71%)	66031 (1.18%)	294427 (1.42%)
Exon sense	42862 (2.18%)	74171 (0.73%)	114131 (2.04%)	255393 (1.23%)
Intron antisense	31838 (1.62%)	95207 (0.94%)	96829 (1.73%)	353679 (1.70%)
Intron sense	57867 (2.95%)	158529 (1.57%)	172485 (3.08%)	484486 (2.33%)
miRNA	1370 (0.07%)	3944255 (39.04%)	1284 (0.02%)	2261930 (10.88%)
rRNA	44028 (2.24%)	742412 (7.35%)	54369 (0.97%)	711225 (3.42%)
Repeat	486909 (24.81%)	1144793 (11.33%)	1528878 (27.30%)	4100141 (19.73%)
snRNA	556 (0.03%)	2371 (0.02%)	627 (0.01%)	1306 (0)
snoRNA	85 (0)	165 (0)	170 (0)	271 (0)
tRNA	3270 (0.17%)	49538 (0.49%)	6303 (0.11%)	58790 (0.28%)
Unannotated sRNAs	1273143 (64.87%)	3818589 (37.80%)	3559384 (63.55%)	12259726 (58.99%)

The clean reads were also mapped against DENV genome to discover DENV encoded small RNA population. We found only 76 reads mapping against DENV genome (Additional file 1).

### Identification of known miRNAs in *Ae. albopictus*

To identify known miRNAs in *Ae. albopictus*, we aligned the small RNAs to the known mature miRNAs and their precursors in miRBase 20.0 to obtain the miRNA count as well as the base bias at the first position. Approximately 1,370 unique sequences (3,944,255 reads) in the control library and 1,284 unique sequences (2,261,930 reads) in the infected library were annotated as miRNA candidates (Table 1). A total of 103 known miRNA genes were identified in the *Ae. albopictus* library (Additional file 2). Most miRNAs ranged in size from 21 to 22 nt, and the base bias at the first position of the identified miRNAs showed a strong preference for ′U′ at the 5′-end, which is coordinated with previous studies (Additional file 3A and B) [17].

We also analyzed miRNA conservations using the available genome assemblies of other insect species, and we confirmed previous study that miR-1889, miR-1890 and miR-1891 were present only in mosquitoes [10]. The conservation of 103 known miRNAs across three other species—*Ae. aegypti*, *Culex quinquefasciatus* and *Anopheles gambiae*—is shown in Additional file 4. Of these 103 miRNAs, all were found in *Ae. aegypti*; 76 were found in all three species; 8 were found in *Ae. aegypti*, but not in *A. gambiae* or *Cx. quinquefasciatus*; 6 were found in *Ae. aegypti* and *A. gambiae*, but not in *Cx. quinquefasciatus*; and 13 were common to *Ae. aegypti* and *Cx. quinquefasciatus*, but were not found in *A. gambiae* (Additional file 3C).

### Identification of novel miRNAs in *Ae. albopictus*

After removing the snRNAs, snoRNAs, rRNAs, tRNAs and known miRNAs, we aligned the remaining unannotated reads against the *Ae. aegypti* genome to predict novel miRNAs in *Ae. albopictus*. A total of 7107 and 23697 potential novel miRNA reads were mapped in control and infected samples. Based on the criteria for miRNAs, 5 novel candidate miRNAs were identified (4 in control and 4 in infected mosquitoes). The miRNAs are shown in Additional file 2, along with their Dicer cleavage site, minimum free energy, frequency of reads and typical secondary structures of the characteristic stem-loop hairpins for the predicted precursors. The secondary structures of the novel candidate miRNAs in *Ae. albopictus* are also shown in Additional file 5.

After that, the remaining unannotated reads were mapped to miRBase 20.0 to predict potential miRNA, a total of 3383 and 19743 predict tags mapped in control and infected library, respectively. As shown in Additional file 6, 3289 and 16353 reads matched to *Bombyx mori* bmo-miR-2796-3p, showed differential expression in control and infected libraries.

### Differentially expressed miRNAs between control and infected *Ae. albopictus*

To compare miRNA abundance in both libraries, we used TPM (tag per million of total RNA reads) to normalize the miRNA expression. And to directly test our sequencing methods’ ability to reproduce miRNA expression levels, a linear regression analysis was performed using TPM of all miRNAs in control and infected mosquitoes. This analysis showed a Pearson correlation coefficient (r) of 0.86, indicating that the two libraries were well correlated.

**Figure 2:**
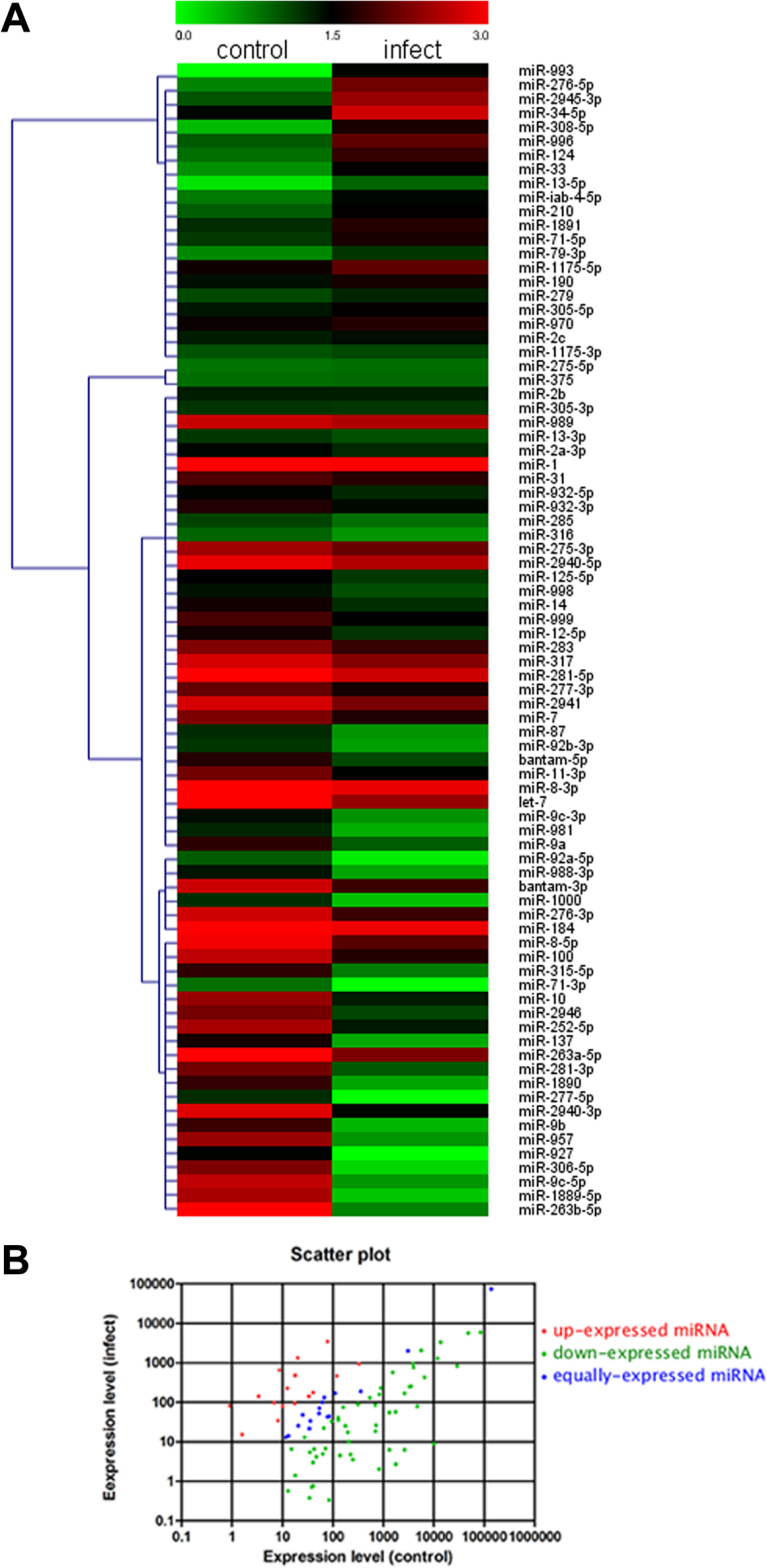
Differential expression of miRNAs in control and infected *Ae. albopictus* mosquitoes. **(A)** Heat map of the miRNAs. Highly expressed miRNAs are indicated in red, and miRNAs with low expression are indicated in green. The absolute signal intensity ranged from 0 to 3. **(B)** Scatter plot of up- and down-regulated miRNAs in control and infected mosquitoes. Each point in the figure represents a miRNA. The X and Y axes show the expression levels in control or infect samples. The red points represent up-expressed miRNAs with a ratio >2, the blue points represent equally-expressed miRNAs with a ratio ≥1/2 and ≤2, and the green points represent down-expressed miRNAs with a ratio <1/2.

**Figure 3:**
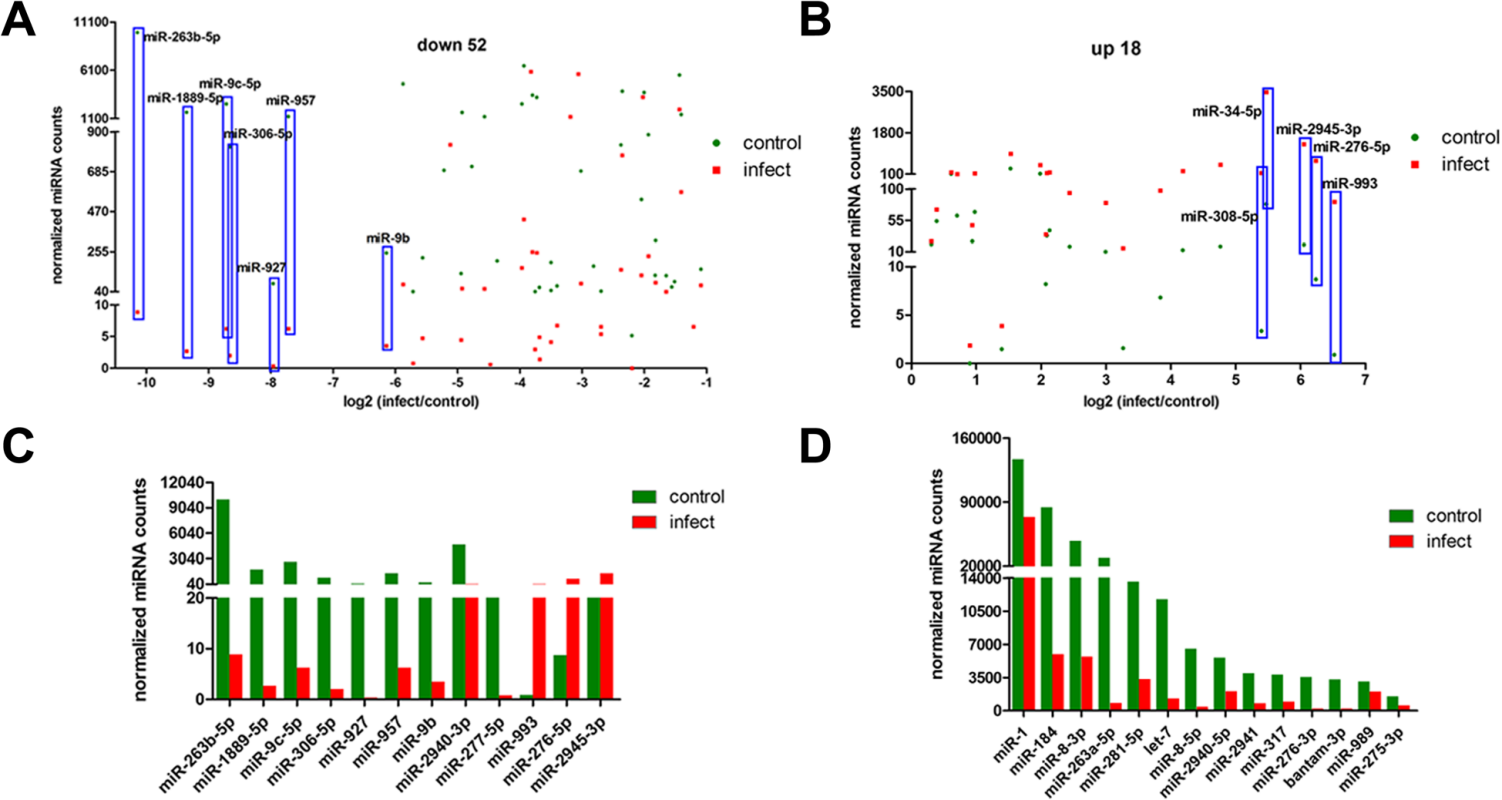
Alteration of miRNAs after dengue virus infection in *Ae. albopictus* mosquitoes. **(A)** Down-regulated miRNAs. The horizontal axis indicates the fold change, and the vertical axis indicates normalized miRNAs expression. Blue indicates the greatly regulated miRNAs. **(B)** Up-regulated miRNAs. **(C)** miRNAs that were down-regulated and up-regulated more than 50-fold. **(D)** The 14 most highly expressed miRNAs in control and infected mosquitoes. T-tests were used for the comparison of miRNAs expression. The P values were performed on the data with the significance threshold selected as 0.05.

After removing the miRNAs with TPM fewer than 10 from the two libraries, 82 known miRNAs were used for the differential expression analysis. Heat map and Scatter Plot revealed different expression patterns of the miRNAs between the control and infected mosquitoes (Figure 2). Based on p-value and log fold change, 66 known miRNAs (15 for up-regulated and 51 for down-regulated) and 4 novel candidate miRNAs (3 for up-regulated and 1 for down-regulated) were differentially expressed (Figure 3A and B). It is noteworthy that most of the miRNAs were down-regulated upon DENV infection. We also identified 9 known miRNAs (miR-263b-5p, miR-1889-5p, miR-9c-5p, miR-306-5p, miR-927, miR-957, miR-9b, miR-2940-3p, and miR-277-5p) to be significantly under-expressed and 3 (miR-993, miR-276-5p, and miR-2945-3p) to be over-expressed than 50-fold (Figure 3C). Moreover, among the 14 miRNAs that were among the top 20 most highly expressed miRNAs in both the control and infected mosquitoes, 12 miRNAs showed higher expression and fold changes (miR-263a-5p, miR-8-5p, miR-184, miR-276-3p, bantam-3p, let-7, miR-8-3p, miR-2941, miR-281-5p, miR-317, miR-2940-5p and miR-275-3p), all of which were significantly down-regulated after infection. miRNAs such as miR-1 and miR-989 were also highly expressed but showed low log2-fold changes (Figure 3D).

### Validation of differentially expressed miRNAs by RT-qPCR

**Figure 4:**
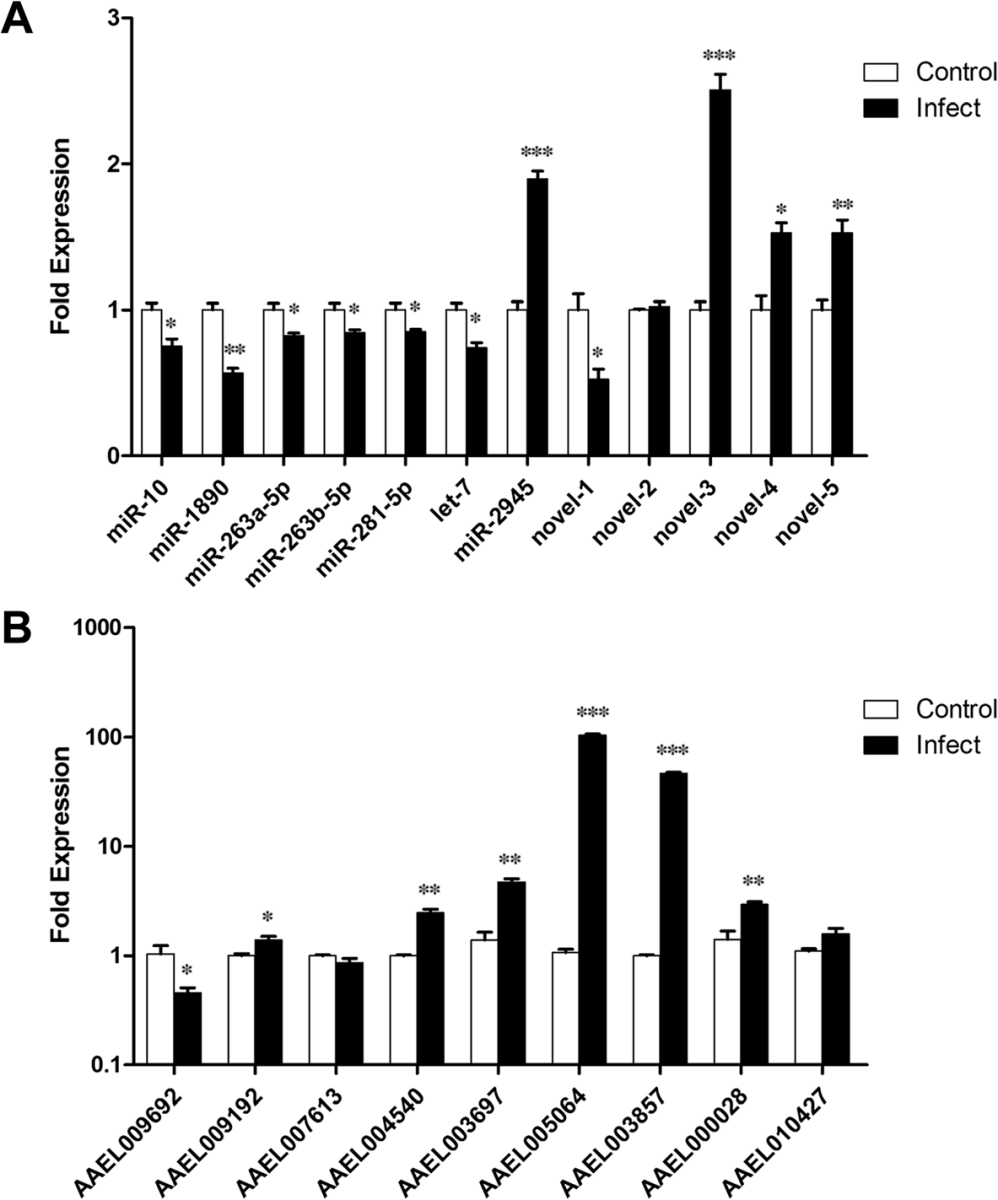
Real Time PCR data analysis of miRNAs and their target mRNAs. **(A)** Expression profiles of 12 miRNAs in female mosquitoes at 7 days after PBS inoculation and DENV-2 inoculation. The relative expression of miRNAs were calculated against control mosquito as a calibrator using 2^-ΔΔCt^ method and normalized to 5S rRNA. **(B)** Expression levels of 9 target mRNAs were detected in control and infected mosquitoes. mRNA levels relative to GAPDH were calculated. T-tests were used for the comparison of RT-qPCR data. Error bars show the standard deviation from three independent experiments with three triplicates each. Asterisks indicate the statistical significance: ***P < 0.001, **P < 0.01, *P < 0.05.

To further validate the expression of differentially expressed miRNAs from high-throughput sequencing, RT-qPCR was performed on five novel candidate miRNAs and seven randomly selected known miRNAs. All of the miRNAs showed consistent expression profiles with the small RNA sequencing data. Results confirmed the down-regulation of 7 miRNAs (miR-10, miR-1890, miR-263a-5p, miR-263b-5p, miR-281-5p, let-7 and novel-1) and the up-regulation of 4 miRNAs (miR-2945, novel-3, novel-4 and novel-5) in infected mosquitoes compared with the uninfected (Figure 4A).

### Prediction of targets for differentially expressed miRNAs

**Figure 5:**
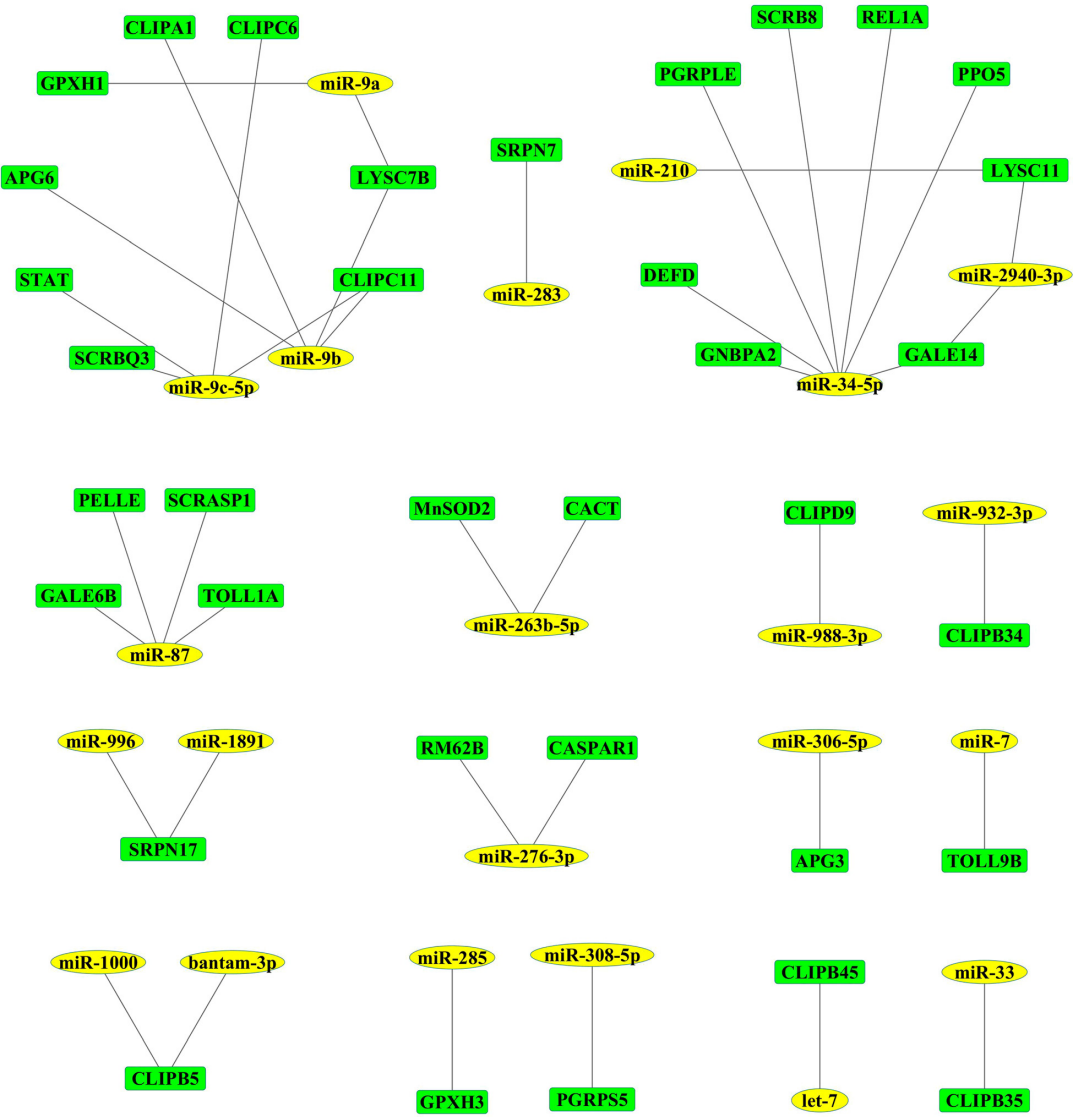
A network of putative interactions between the differentially expressed miRNAs and the immune target genes. Yellow indicates miRNAs; green indicates immune target genes.

To investigate the regulated miRNAs functions, we predicted the potential targets of the 66 differentially expressed known miRNAs in *Ae. albopictus* upon DENV-2 infection. A total of 1421 genes were predicted, including 35 immune transcripts, such as Toll-like receptor (TOLLs), clip domain serine protease (CLIPs), serine protease inhibitors (SRPNs), scavenger receptors (SCRs), and so on. The network of interaction between miRNAs and immune targets was shown in Figure 5. Nine immune target genes were randomly selected to perform RT-qPCR in control and DENV2-injected mosquitoes. As a result, 6 targets (AAEL000028, AAEL004540, AAEL005064, AAEL009192, AAEL003697, AAEL003857) were up-regulated, AAEL009692 was down-regulated, and 2 target genes (AAEL007613, AAEL010427) were non-regulated (Figure 4B).

**Figure 6:**
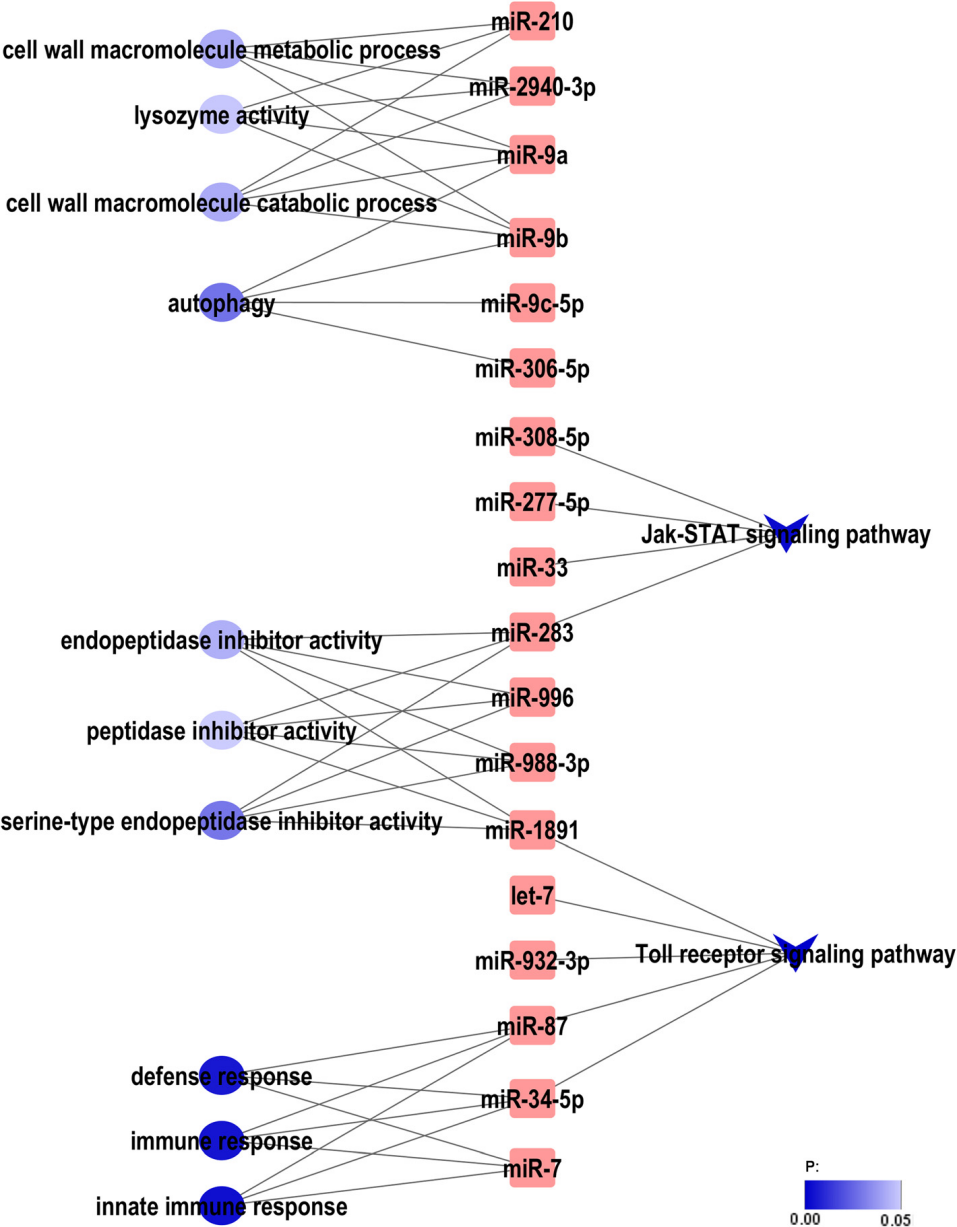
GO and Pathway analysis of the differentially expressed miRNAs. Round Rectangle indicates miRNAs; Ellipse indicates GO classes; V indicates pathways.

To further study the potential roles of the regulated miRNAs in DENV-*Ae. albopictus* interactions, we performed GO enrichment and pathway analysis on the top three targets of the differentially expressed miRNAs (Additional file 7) using DAVID Bioinformatics software [18]. Ten GO terms were found to be enriched, including three immune terms (“innate immune response”, “immune response”, and “defense response”). Based on KEGG analysis, many target genes of miRNAs were involved in Jak-STAT signaling pathway and Toll receptor signaling pathway. Taken the KEGG and GO analysis together, miR-34-5p and miR-87 were found to be related to immune pathway (Toll-like receptor signaling pathway) and immune GO terms (defense response, immune response and innate immune response) (Figure 6).

## Discussions

MiRNAs have been shown to be involved in the mosquito immune responses [19-22]. In this study, we aim to identify miRNAs involved in the response to DENV-2 in *Ae. albopictus* using high-throughput sequencing technology. We obtained about 10 and 20 million reads from control and infected mosquitoes, but more than 60% of reads in each of the sequencing libraries were not annotated. It may be attributed to the differences between *Ae. albopictus* and *Ae. aegypti* genome.

We identified 103 known and 5 novel candidate miRNAs in *Ae. albopictus*. Previous studies showed that, pathogen infection can influence the expression of mosquito miRNAs. In *Ae. aegypti*, distinct miRNA expression profiles were observed after CHIKV infection, WNV infection and *Wolbachia* infection [23-25]. Likewise, *Plasmodium* also changed the miRNAs expression in *A. gambiae* [11]. In this study, we found that 66 known miRNAs displayed different expressions during DENV infection. Previous reports showed that, miR-1889-5p and miR-9c-5p were down-regulated in *Wolbachia* and DENV infected *Ae. aegypti*, respectively [24,26]. miR-989, which was down-regulated in WNV infected *Cx. quinquefasciatus* and *Wolbachia* infected *Ae. aegypti*, has been reported to play roles in mediating flavivirus infection in the mosquito host [12,14]. In our study, miR-989, miR-1889-5p and miR-9c-5p were also down-regulated following DENV infection. These results indicate the possibility that, as observed in other mosquitoes, miRNAs likely participate in host-virus interaction in *Ae. albopictus*.

In mosquitoes, a large number of miRNAs have been reported to regulate the host immune responses. miR-2940-5p, which is highly induced in *Wolbachia*-infected *Ae. aegypti*, was previously reported to enhance *Wolbachia* efficient maintenance and limit replication of DENV in *Ae. aegypti* [13]. Another study demonstrated that miR-2940-5p is down-regulated in *Ae. albopictus* C6/36 cells in response to WNV infection and restrict WNV replication [25]. In addition, miR-2940-5p and miR-2940-3p were reported decreased in CHIKV- infected *Ae. albopictus* [23]*.* In this study, we found that, miR-2940-5p and miR-2940-3p were significantly down-regulated upon DENV-2 infection. These studies indicate that miR-2940-5p and miR-2940-3p may also be involved in interactions between *Ae. albopictus* mosquito hosts and DENV.

We have further predicted targets of the 66 modulated miRNAs, and note that some differentially expressed miRNAs target a number of immune genes. Then we chose the top three targets of each miRNA to perform GO enrichment and pathways analysis. Taken the GO and KEGG analysis together, we found miR-34-5p and miR-87 to be related to immune GO terms and immune pathway. The down-regulated miR-87, which was reported previously, decreased upon CHIKV infection in *Ae. albopictus* mosquitoes [23], targeted the TOLL pathway signalling Ser/Thr Kinase (PELLE, AAEL006571), Toll-like receptor (TOLL1A, AAEL007613), Class A Scavenger Receptor with Serine Protease domain (SCRASP1, AAEL009192) and galectin (GALE6B, AAEL012003). The up-regulated miR-34-5p, targeted antimicrobial peptides defensin D (DEFD, AAEL003857), Peptidoglycan Recognition Protein LE (PGRP-LE, AAEL013112), and other immune transcripts. Another target of miR-34-5p, the TOLL pathway signalling NF-kappaB Relish-like transcription factor (REL1, AAEL007696), has been reported in another study as the activation of Rel1 leads to a decreased DENV titer, while repression of Rel1 activation leads to a significant increase in DENV replication [27]. Mosquitoes rely on their effective innate immune system to limit pathogen infections. Studies that were mainly conducted in the *Ae. aegypti* have shown that, immune responses are largely regulated by three major immune signaling pathways: the Toll, Imd, and JAK-STAT pathways [28]. The Toll pathway has been shown to direct anti-dengue defenses in the *A. aegypti* [27,29-31]. In this study, based on KEGG analysis, we found miR-34-5p and miR-87 may be related to the Toll-like receptor signaling pathway. And the GO analysis showed miR-34-5p and miR-87 may be involved in the defense response, immune response and innate immune response. Taken together, these studies indicate that miR-34-5p and miR-87 may be associated with the anti-pathogen and immune responses.

## Conclusion

In conclusion, we identified miRNAs and evaluated their expression patterns in *Ae. albopictus* upon DENV-2 infection using Illumina sequencing. Sixty-six known differentially expressed miRNAs and 5 novel candidate miRNAs were identified. Target prediction and functional analysis of these miRNAs suggested that miR-34-5p and miR-78 might be involved in anti-pathogen and immune responses. This study provides important information for studying the interaction between DENV and *Ae. albopictus* miRNAs, and it also provides clues for further studies on the mechanisms of immune responses in DENV-infected *Ae. albopictus*.

## Methods

### Ethics statement

All vertebrate animals were housed and handled in strict accordance with the guidelines of the institutional and National Committees of Animal Use and Protection. All experimental procedures involving mice were approved by the Committee on the Ethics of Animal Experiments of Southern Medical University (Permit Number: SCXK 2006-0015).

### Cell culture maintenance and mosquito rearing

The C6/36 *Ae. albopictus* cell line and the DENV-2 strain NGC were kindly provided by the Department of Parasitology, Zhongshan School of Medicine, Sun Yat-sen University. C6/36 (*Ae. albopictus*) cells were cultured in Dulbecco's modified Eagle’s medium (DMEM, Invitrogen) supplemented with 10% fetal calf serum (FBS) (GIBCO), 2 mM L-glutamine, 50 U/mL penicillin and 50 μg/L streptomycin and maintained at 28°C.

The *Aedes albopictus* strain was obtained from the CDC of Guangdong Province, PRC, which was isolated from Foshan, Guangdong, China and established in the laboratory since 1981. Mosquitoes were maintained at 27 ± 1°C, with 70–80% relative humidity and a 12:12 hour light-dark cycle. Larvae were fed with yeast powder, and adults were fed with a 10% glucose solution. Adult female mosquitoes were fed with the blood of an anesthetized mouse, which was then returned to the animal room.

### DENV-2 infections

The C6/36 cells were infected with DENV-2 virus (New Guinea C strain) at a multiplicity of infection (MOI) of 5 at 37°C in 5% CO_2_ for 1 h, and harvested at 7 days post-infection (dpi). Then culture supernatants were collected to determine the titer of infectious virus by RT-qPCR, plaque assay and Karber method [32-36]. Results showed the virus titer is 10^6.85^ plaque-forming units (PFU)/ml and 7 log_10_TCID50/mL (50% tissue culture infective dose, TCID50). Viral RNA copies were calculated as 10^9^ copies/μg using the standard curve method.

For mosquito infection through intra-thoracic inoculation, 0.2 μL of the above virus suspension (10^6.85^ PFU/ml) was used for inoculation into each female; PBS was used as the control. Then, the mosquitoes were maintained at (27 ± 1) °C with 70–80% humidity and supplied with 10% glucose solution. Pools of 14 whole mosquitoes were harvested at 7 dpi from both DENV2-injected and control mosquitoes. Infection rate, as determined by plaque assay [26,37,38], was 100% at 7 dpi.

### RNA isolation and deep sequencing

Pools of 14 whole mosquitoes were ground together to a fine powder in liquid nitrogen at 7 dpi. Total RNA was extracted from 14 whole mosquitoes using TRIzol Reagent (Invitrogen) according to manufacturer's instructions. RNAs ranging from 18 to 30 nt were then purified from a 15% denaturing polyacrylamide gel and sequentially ligated to proprietary adapters according to the manufacturer’s instructions (Illumina Inc) before sequencing. The gel-purified ligation products were converted to cDNA and amplified using RT-PCR with 18 PCR cycles to produce libraries that were then sequenced using the Illumina Genome Analyzer (Illumina, San Diego, CA, USA) at Huada Genomics Institute Co. Ltd, Shenzhen, China. Raw sequencing data have been submitted to the National Center for Biotechnology Information (NCBI) (accession number SRA129084).

### Bioinformatics

After removing the adapter sequences and low-quality sequences, tags with lengths ranging from 18 to 30 nt were screened against the GenBank database (http://www.ncbi.nlm.nih.gov/genbank/) and Rfam database (http://www.sanger.ac.uk/software/Rfam) to remove rRNA, tRNA, snRNA, snoRNA, repeats, exon sequences and intron sequences. Considering that no miRNA information for *Ae. albopictus* was in miRBase 20.0, the remaining sequencing reads were aligned to search all known precursor/mature miRNAs of *Ae. aegypti* in miRBase 20.0 [39]. Only perfectly or near-perfectly (1–2 mismatches) matching sequences were considered to represent conserved miRNAs, and each miRNA at least 15 reads to be considered expressed. Finally, to discover potential miRNAs, all unannotated reads were aligned against the *Ae. aegypti* genome using SOAP [40]. RNA secondary structures were predicted using RNAfold (http://rna.tbi.univie.ac.at/cgi-bin/RNAfold.cgi) and analyzed using MIPRED and MIREAP (http://sourceforge.net/projects/mireap/). The identified stem-loop hairpins fulfilled three criteria: mature miRNAs were present in one arm of the hairpin precursors, which also lacked large internal loops or mismatches; the secondary structures of the hairpins were stable, with free energies of hybridization lower than -20 kcal/mol; and hairpins were located in intergenic regions or introns. Subsequently, miRAlign was adopted to filter mature miRNA genes and predict new miRNA genes, and each novel miRNA in the samples was required to have at least 15 reads to be considered as expressed. The whole process is shown as Additional file 8.

### Expression profiling of miRNAs in response to DENV

All miRNA expression data were normalized using tag per million of total RNA reads (TPM) to compare miRNA abundance in both libraries. TPM = (The count of the miRNA in a particular sample)/(Total miRNA counts from this sample) × 1 million. Because extremely low abundances might not accurately reflect their true abundance, the known miRNAs with TPM less than 10 in the two libraries were removed from the differential expression analysis. Changes in known miRNA expression in infected versus control samples were considered significant when their p values were below 0.05. miRNAs with log2-fold changes higher than 1 were designated as significantly up-regulated, and those with log2-fold changes less than -1 were designated as significantly down-regulated.

### Target prediction of microRNAs and functional analysis

RNAhybrid and SMVLight were used to predict the targets of the miRNAs (p value < 0.05, Prediction-value > 1, a minimum free energy (mfe) < -20.0 kcal/mol, one G:U mismatch was permitted in the seed sequence, and a maximum of three G:U mismatches were allowed in the flanking sequence).

The vectorbase immune-related targets (https://www.vectorbase.org/) were then used to generate the miRNA:mRNA network. Cytoscape was used for visualizing the networks [41].

Then, the top three targets of the differentially expressed miRNAs were performed GO enrichment and pathway analysis using the DAVID Bioinformatics Resources 6.7 (http://david.abcc.ncifcrf.gov/).

### RT-qPCR

Real time PCR analysis was performed on known miRNAs and target genes using the miScript PCR system, including the miScript Reverse Transcription Kit (Qiagen, Duesseldorf, Germany) and the miScript SYBR Green PCR kit (Qiagen). Amplification was carried out using MX3005P TM Real Time PCR System (Stratagene, CA, USA). Real time PCR analysis was also performed on novel candidate miRNAs using a stem-loop-mediated reverse transcription real-time PCR method. Expression levels of novel miRNAs were analyzed using a 7500 Real-Time PCR system (Applied Biosystems). All reactions were run in triplicate. 5S rRNA and GAPDH were used as endogenous control for miRNA and DENV-2, respectively. All the qPCR primers used in this study are shown in Additional file 9. Expression levels were then calculated against control mosquito as a calibrator using 2^-ΔΔCt^ method.

### Statistical analysis

All quantitative RT-qPCR results were representative of at least three independent experiments, each with three technical replicates. Statistical tests were performed with SPSS 22.0 (SPSS, Chicago, IL). T-tests were used for the comparison of RT-qPCR data. The P values were performed on the data with the significance threshold selected as 0.05. Asterisks indicate the statistical significance: ***P < 0.001, **P < 0.01, *P < 0.05. Error bars show the standard deviation.

## Additional files

Additional files 1:
**The sequencing reads matched to the DENV genome.**
Additional files 2:
**Known and Novel candidate miRNAs identified in** ***Aedes albopictus.***
Additional files 3:
**Analysis of first nucleotide bias and conservation of known miRNAs.** The first nucleotide bias of miRNAs in control mosquitoes **(A)** and infected mosquitoes **(B)**. The horizontal axis indicates the length of miRNAs in *Ae. albopictus*, and the vertical axis indicates the frequency (%). **(C)** The number of miRNAs that were conserved across *Ae. aegypti*, *Cx. quinquefasciatus* and *A. gambia*e. Additional files 4:
**Family analysis of the known miRNAs in** ***Aedes albopictus.***
Additional files 5:
**The secondary structures of novel candidate miRNAs in** ***Aedes albopictus.***
Additional files 6:
***Aedes albopictus*** **small RNA sequences match known** ***Bombyx mori*** **miR-2796-3p.** Abbreviations: nt, length of small RNA read in nucleotides; N, number of reads in each sample that showed the exact sequence; Nucleotides in red background indicate sequence same between *Ae. albopictus* and *Bombyx mori*. Additional files 7:
**The top three predicted targets of the differentially expressed known miRNAs.**
Additional files 8:
**The whole process of bioinformatics.**
Additional files 9:
**qPCR primers used in this study.**

## Abbreviations

DENV: Dengue virus
*Ae. albopictus*: *Aedes albopictus*
miRNAs: microRNAs
*Cx. quinquefasciatus*: *Culex quinquefasciatus*
*A. gambiae*: *Anopheles gambiae*
*Ae. aegypti*: *Aedes aegypti*
TPM: Tag per million of total RNA reads
RT-qPCR: Reverse transcription-quantitative polymerase chain reaction

## References

[1] Bian G, Zhou G, Lu P, Xi Z (2013). Replacing a native *Wolbachia* with a novel strain results in an increase in endosymbiont load and resistance to dengue virus in a mosquito vector. PLoS Negl Trop Dis.

[2] Bäck AT, Lundkvist Å. Dengue viruses-an overview. Infection Ecology & Epidemiology. 2013;3.doi:10.3402/iee.v3i0.19839. http://www.ncbi.nlm.nih.gov/pubmed/24003364. 10.3402/iee.v3i0.19839 PMC3759171 24003364

[3] Mackenzie JS, Gubler DJ, Petersen LR (2004). Emerging flaviviruses: the spread and resurgence of Japanese encephalitis, West Nile and dengue viruses. Nat Med.

[4] Chen X (2009). Small RNAs and their roles in plant development. Annu Rev Cell Dev Biol.

[5] Smibert P, Lai EC (2008). Lessons from microRNA mutants in worms, flies and mice. Cell Cycle.

[6] Bushati N, Cohen SM (2007). microRNA functions. Annual Review of Cell and Dev Biol.

[7] Carrington JC (2003). Role of MicroRNAs in plant and animal development. Science.

[8] Wang X, Zhang J, Li F, Gu J, He T, Zhang X (2005). MicroRNA identification based on sequence and structure alignment. Bioinformatics.

[9] Mead E, Tu Z (2008). Cloning, characterization, and expression of microRNAs from the Asian malaria mosquito, *Anopheles stephensi*. BMC Genomics.

[10] Li S, Mead EA, Liang S, Tu Z (2009). Direct sequencing and expression analysis of a large number of miRNAs in *Aedes aegypti* and a multi-species survey of novel mosquito miRNAs. BMC Genomics.

[11] Winter F, Edaye S, Huttenhofer A, Brunel C (2007). *Anopheles gambiae* miRNAs as actors of defence reaction against *Plasmodium* invasion. Nucleic Acids Res.

[12] Skalsky RL, Vanlandingham DL, Scholle F, Higgs S, Cullen BR (2010). Identification of microRNAs expressed in two mosquito vectors, *Aedes albopictus* and *Culex quinquefasciatus*. BMC Genomics.

[13] Zhang G, Hussain M, O'Neill SL, Asgari S (2013). *Wolbachia* uses a host microRNA to regulate transcripts of a methyltransferase, contributing to dengue virus inhibition in *Aedes aegypti*. Proc Natl Acad Sci.

[14] Hussain M, Frentiu FD, Moreira LA, O'Neill SL, Asgari S (2011). *Wolbachia* uses host microRNAs to manipulate host gene expression and facilitate colonization of the dengue vector *Aedes aegypti*. Proc Natl Acad Sci.

[15] Hussain M, Walker T, O'Neill SL, Asgari S (2013). Blood meal induced microRNA regulates development and immune associated genes in the Dengue mosquito vector, *Aedes aegypti*. Insect Biochem Mol Biol.

[16] Thirugnanasambantham K, Hairul-Islam VI, Saravanan S, Subasri S, Subastri A (2013). Computational approach for identification of *Anopheles gambiae* miRNA involved in modulation of host immune response. Appl Biochem Biotechnol.

[17] Sun J, Li M, Li Z, Xue J, Lan X, Zhang C (2013). Identification and profiling of conserved and novel microRNAs from Chinese Qinchuan bovine longissimus thoracis. BMC Genomics.

[18] Huang DW, Sherman BT, Lempicki RA (2008). Systematic and integrative analysis of large gene lists using DAVID bioinformatics resources. Nat Protoc.

[19] Hussain M, Asgari S (2014). MicroRNAs as mediators of insect host-pathogen interactions and immunity. J Insect Physiol.

[20] Asgari S (2014). Role of microRNAs in Arbovirus/Vector Interactions. Viruses.

[21] Asgari S (2013). MicroRNA functions in insects. Insect Biochem Mol Biol.

[22] Lucas KJ, Myles KM, Raikhel AS (2013). Small RNAs: a new frontier in mosquito biology. Trends Parasitol.

[23] Shrinet J, Jain S, Jain J, Bhatnagar RK, Sunil S (2014). Next generation sequencing reveals regulation of distinct *Aedes* microRNAs during chikungunya virus development. PLoS Negl Trop Dis.

[24] Mayoral JG, Etebari K, Hussain M, Khromykh AA, Asgari S (2014). *Wolbachia* infection modifies the profile, shuttling and structure of MicroRNAs in a mosquito cell line. PLoS One.

[25] Slonchak A, Hussain M, Torres S, Asgari S, Khromykh AA (2014). Expression of mosquito MicroRNA Aae-miR-2940-5p is downregulated in response to West Nile Virus infection to restrict viral replication. J Virol.

[26] Campbell CL, Harrison T, Hess AM, Ebel GD (2014). MicroRNA levels are modulated in *Aedes aegypti* after exposure to Dengue-2. Insect Mol Biol.

[27] Ramirez JL, Dimopoulos G (2010). The Toll immune signaling pathway control conserved anti-dengue defenses across diverse Ae aegypti strains and against multiple dengue virus serotypes. Developmental & Comparative Immunology.

[28] Souza-Neto JA, Sim S, Dimopoulos G (2009). An evolutionary conserved function of the JAK-STAT pathway in anti-dengue defense. Proc Natl Acad Sci.

[29] Pan X, Zhou G, Wu J, Bian G, Lu P, Raikhel AS (2012). *Wolbachia* induces reactive oxygen species (ROS)-dependent activation of the Toll pathway to control dengue virus in the mosquito *Aedes aegypti*. Proc Natl Acad Sci.

[30] Luplertlop N, Surasombatpattana P, Patramool S, Dumas E, Wasinpiyamongkol L, Saune L (2011). Induction of a peptide with activity against a broad spectrum of pathogens in the *Aedes aegypti* salivary gland, following infection with dengue virus. PLoS Pathog.

[31] Xi Z, Ramirez JL, Dimopoulos G (2008). The *Aedes aegypti* toll pathway controls dengue virus infection. PLoS Pathog.

[32] Salazar MI, Richardson JH, Sánchez-Vargas I, Olson KE, Beaty BJ (2007). Dengue virus type 2: replication and tropisms in orally infected *Aedes aegypti* mosquitoes. BMC Microbiol.

[33] Keene KM, Foy BD, Sanchez-Vargas I, Beaty BJ, Blair CD, Olson KE (2004). RNA interference acts as a natural antiviral response to O'nyong-nyong virus (*Alphavirus*; Togaviridae) infection of *Anopheles gambiae*. Proc Natl Acad Sci.

[34] Gargan TN, Bailey CL, Higbee GA, Gad A, El SS (1983). The effect of laboratory colonization on the vector-pathogen interactions of Egyptian *Culex pipiens* and Rift Valley fever virus. Am J Trop Med Hyg.

[35] Tay MYF, Saw WG, Zhao Y, Chan KWK, Singh D, Chong Y (2015). The C-terminal 50 amino acid residues of dengue NS3 protein are important for NS3-NS5 interaction and viral replication. J Biol Chem.

[36] Karber G (1931). Beitrag zur kollektiven Behandlung pharmakologischer Reihenversuche. Arch Exp Pathol Parmakol.

[37] Hess AM, Prasad AN, Ptitsyn A, Ebel GD, Olson KE, Barbacioru C (2011). Small RNA profiling of Dengue virus-mosquito interactions implicates the PIWI RNA pathway in anti-viral defense. BMC Microbiol.

[38] Campbell CL, Keene KM, Brackney DE, Olson KE, Blair CD, Wilusz J (2008). *Aedes aegypti* uses RNA interference in defense against Sindbis virus infection. BMC Microbiol.

[39] Griffiths-Jones S, Saini HK, van Dongen S, Enright AJ (2007). miRBase: tools for microRNA genomics. Nucleic Acids Res.

[40] Li R, Li Y, Kristiansen K, Wang J (2008). SOAP: Short Oligonucleotide Alignment Program. Bioinformatics.

[41] Shannon P (2003). Cytoscape: a software environment for integrated models of biomolecular interaction networks. Genome Res.

