# Supplementary figures and images for: The expression profile of Aedes albopictus miRNAs is altered by dengue virus serotype-2 infection
Source: Cell Biosci. 2015 Apr 16;5:16. doi: 10.1186/s13578-015-0009-y (PMC4411651; doi:10.1186/s13578-015-0009-y)

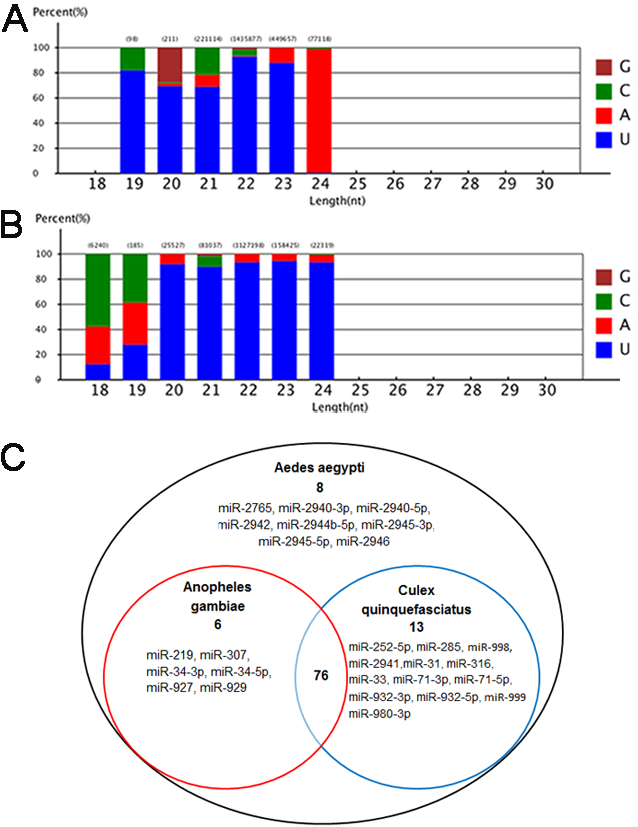

Supplement: Additional files 3: — Analysis of first nucleotide bias and conservation of known miRNAs. The first nucleotide bias of miRNAs in control mosquitoes (A) and infected mosquitoes (B). The horizontal axis indicates the length of miRNAs in Ae. albopictus, and the vertical axis indicates the frequency (%). (C) The number of miRNAs that were conserved across Ae. aegypti, Cx. quinquefasciatus and A. gambiae. [file 13578_2015_9_MOESM3_ESM.tiff]

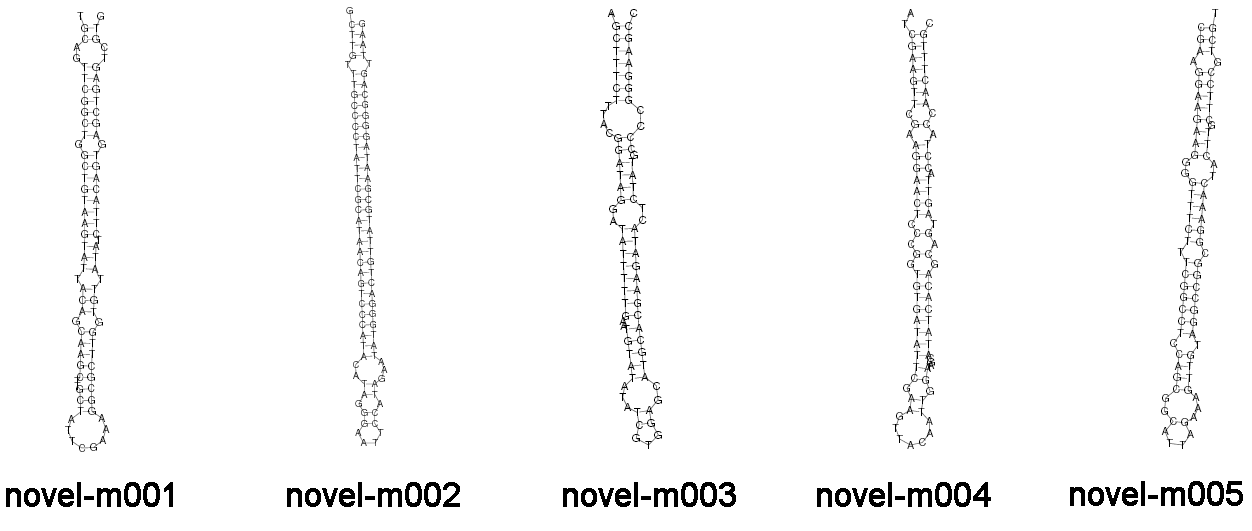

Supplement: Additional files 5: — The secondary structures of novel candidate miRNAs in Aedes albopictus. [file 13578_2015_9_MOESM5_ESM.tiff]

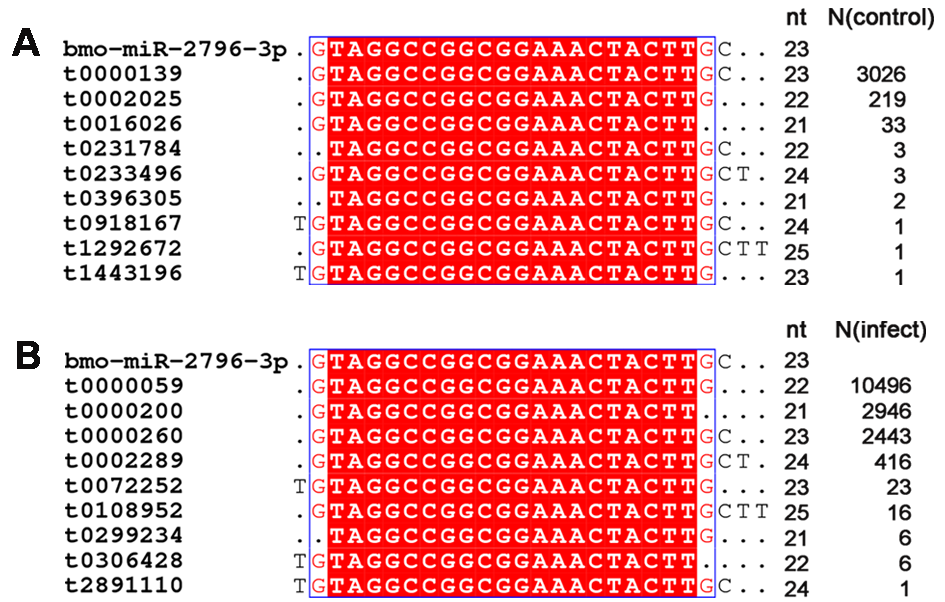

Supplement: Additional files 6: — Aedes albopictus small RNA sequences match known Bombyx mori miR-2796-3p. Abbreviations: nt, length of small RNA read in nucleotides; N, number of reads in each sample that showed the exact sequence; Nucleotides in red background indicate sequence same between Ae. albopictus and Bombyx mori. [file 13578_2015_9_MOESM6_ESM.tiff]

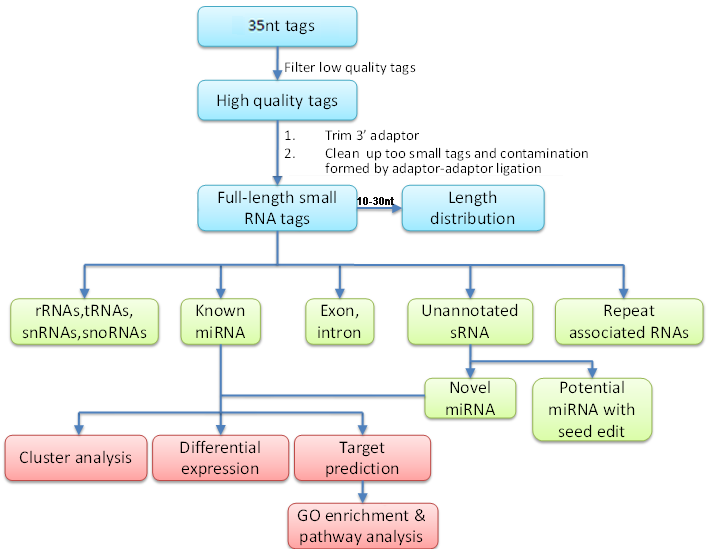

Supplement: Additional files 8: — The whole process of bioinformatics. [file 13578_2015_9_MOESM8_ESM.tiff]
